# Comprehensive Analysis of Peripheral Exosomal circRNAs in Large Artery Atherosclerotic Stroke

**DOI:** 10.3389/fcell.2021.685741

**Published:** 2021-06-21

**Authors:** Qi Xiao, Ruihua Yin, Yuan Wang, Shaonan Yang, Aijun Ma, Xudong Pan, Xiaoyan Zhu

**Affiliations:** ^1^Department of Neurology, The Affiliated Hospital of Qingdao University, Qingdao, China; ^2^Institute of Cerebrovascular Diseases, The Affiliated Hospital of Qingdao University, Qingdao, China; ^3^Department of Critical Care Medicine, The Affiliated Hospital of Qingdao University, Qingdao, China

**Keywords:** large artery atherosclerotic stroke, exosomes, circular RNAs, ceRNA network, RNA sequencing

## Abstract

Exosomes are crucial vehicles in intercellular communication. Circular RNAs (circRNAs), novel endogenous noncoding RNAs, play diverse roles in ischemic stroke. Recently, the abundance and stability of circRNAs in exosomes have been identified. However, a comprehensive analysis of exosomal circRNAs in large artery atherosclerotic (LAA) stroke has not yet been reported. We performed RNA sequencing (RNA-Seq) to comprehensively identify differentially expressed (DE) exosomal circRNAs in five paired LAA and normal controls. Further, quantitative real-time PCR (qRT-PCR) was used to verify the RNA-Seq results in a cohort of stroke patients (32 versus 32). RNA-Seq identified a total of 462 circRNAs in peripheral exosomes; there were 25 DE circRNAs among them. Additionally, circRNA competing endogenous RNA (ceRNA) network and translatable analysis revealed the potential functions of the exosomal circRNAs in LAA progression. Two ceRNA pathways involving 5 circRNAs, 2 miRNAs, and 3 mRNAs were confirmed by qRT-PCR. In the validation cohort, receiver operating characteristic (ROC) curve analysis identified two circRNAs as possible novel biomarkers, and a logistic model combining two and four circRNAs increased the area under the curve compared with the individual circRNAs. Here, we show for the first time the comprehensive expression of exosomal circRNAs, which displayed the potential diagnostic and biological function in LAA stroke.

## Introduction

Stroke is a global health problem, the third commonest cause of death; approximately 87% of stroke is ischemic stroke (Capodanno et al., 2016; Vos et al., 2020). Large artery atherosclerotic (LAA) stroke is the most common subtype of ischemic stroke (Adams et al., 1993; Shen et al., 2019; Wu et al., 2019). Although previous studies have identified some candidate blood biomarkers for the diagnosis and prognosis of stroke, such as miRNAs, circular RNAs (circRNAs), and lncRNAs (Zhang X. et al., 2019), researchers have found that RNAs in exosomes are more enriched and stable, leading to effective membrane protection (Li et al., 2015; Hu et al., 2018; Yu and Chen, 2019).

Exosomes are extracellular vesicles, 40–160 nm in diameter, that are secreted by almost all cells and carry molecular cargoes, including RNA species (such as circRNAs, miRNAs, and mRNAs), DNAs, proteins, and lipids (Kalluri and LeBleu, 2020). Exosomes play diverse roles in intercellular communication, acting as loading and delivery systems and causing therapeutic effects (van Niel et al., 2018; Kalluri and LeBleu, 2020). Atherosclerosis is the principal cause of LAA stroke (Libby et al., 2016). Importantly, exosomes have been reported to have multifaceted functions in the process of atherosclerosis (Chen et al., 2020; Wang H. et al., 2020). In addition, our previous studies have found that exosomal miR-145 derived from mesenchymal stem cells (MSCs) protects against atherosclerosis (AS) development (Yang et al., 2020).

circRNAs are a type of noncoding RNA that lack 5′ caps and 3′ polyadenylation (polyA) tails and have a closed loop structure produced in eukaryotic cells by back-splicing reactions (Li et al., 2018). circRNAs have recently emerged as potential biomarkers and therapeutic candidates with tissue specificity and are particularly abundant in peripheral blood and tissues, such as the brain, vascular smooth muscle cells, and human umbilical vein cells (Chen, 2020). Until now, no studies have reported the comprehensive expression of circRNAs within exosomes present in peripheral blood during LAA stroke.

In the present study, we isolated exosomes from peripheral blood circulation and performed comprehensive expression profiling of circRNAs. Our findings may provide novel perspectives on the biological function of exosomal circRNAs in LAA stroke.

## Materials and Methods

### Study Participants

Patients with LAA ischemic stroke (LAA, *n* = 37) and normal controls (NC, *n* = 37) were recruited within 72 h after symptom onset from the Affiliated Hospital of Qingdao University. The enrolment period was February 2020 to July 2020. All subjects were diagnosed with LAA stroke with an acute focal neurological deficit, a new infarction on MRI or CT, and atherosclerotic stenosis (≥50% stenosis in intracranial or extracranial arteries) according to the TOAST criteria (Adams et al., 1993). Subjects were excluded if they had other subtypes of stroke, thrombolysis, serious heart disease (acute myocardial infarction, atrial fibrillation, etc.), serious nephrosis or liver disease, abscess, or tumors. Subjects were matched on age, sex, body mass index (BMI), hypertension, diabetes, smoking and drinking. Participants randomly selected five pairs for sequencing and the other 32 pairs for the validation phase. The study protocol was approved by the Ethical Committee of the Affiliated Hospital of Qingdao University. All participants provided informed consent.

### Exosome Isolation

Total exosome isolation (from plasma) reagent (Invitrogen, Cat 4484450, Carlsbad, United States) was used (Tian et al., 2020). Briefly, plasma samples were centrifuged at 2,000 × *g* for 20 min at room temperature to remove cells and debris and then centrifuged at 10,000 × *g* for 20 min a second time to remove debris. Then, 1 mL plasma was added to 0.5 mL PBS. The sample was mixed thoroughly by vortexing; then, 50 μL of Proteinase K was added to the mixture, and the mixture was incubated at 37°C for 10 min. Next, 300 μL of exosome precipitation reagent was added to the supernatant. After mixing, the mixture was incubated at 4°C for 30 min and then centrifuged at 10,000 × *g* for 5 min. Exosomes were contained in the pellet at the bottom of the tube, and the pellet was resuspended in PBS.

### Transmission Electron Microscopy

The resuspended exosome solution (10 μL) was placed on a copper grid for 2 min at room temperature. Then, the exosomes were placed in 2% phosphotungstic acid for 2 min and washed with sterile distilled water. The morphology of the exosomes was observed using Transmission Electron Microscopy (TEM) (Hitachi, Tokyo, Japan) (Zhang J. T. et al., 2019).

### Nanoparticle Tracking Analysis

The exosome pellets were resuspended in 1 mL PBS, examined using a ZetaView PMX 110 instrument (Particle Metrix, Meerbusch, Germany) and analyzed using nanoparticle tracking analysis (NTA) software (ZetaView 8.04.02) to determine the particle size and quantity (Min et al., 2019).

### Western Blotting

Total proteins of the exosomes were extracted with standard RIPA buffer with PMSF (MCE, United States) at a volume ratio of 99:1, and the concentration of protein was normalized using the BCA assay. The protein samples (25 μg) were then subjected to 10% SDS-PAGE and transferred onto a membrane. The PVDF membrane was incubated with primary antibodies at 4°C overnight, including CD9, CD63, TSG101, and GRP94 (Abcam, ab92726, ab134045, ab125011, and ab238126 Cambs, United Kingdom), and then, the membrane was incubated with HRP-conjugated secondary antibodies (Abcam, Cambs, United Kingdom) for 1 h (Min et al., 2019).

### Library Preparation and Sequencing

A total amount of 5 μg RNA per sample was used as input material for the RNA sample preparations. Sequencing libraries were generated by the NEBNextR Ultra Directional RNA Library Prep Kit for Illumina R (NEB, United States) following the manufacturer’s recommendations. First-strand cDNA was synthesized using random hexamer primers and M-MuLV Reverse Transcriptase (RNaseH). Second-strand cDNA synthesis was subsequently performed using DNA polymerase I and RNase H. After adenylation of the 3′ ends of the DNA fragments, NEBNext adaptors with hairpin loop structures were ligated to prepare for hybridization. To preferentially select cDNA fragments of 150∼200 bp in length, the library fragments were purified with the AMPure XP system (Beckman Coulter, Beverly, United States). Then, PCR was performed with Phusion High-Fidelity DNA polymerase, universal PCR primers and Index (X) Primer. Finally, the products were purified (AMPure XP system), and the library quality was assessed using the Agilent Bioanalyser 2100 system.

Clustering of the index-coded samples was performed using a cBot Cluster Generation System using TruSeq PE Cluster Kit v3-cBot-HS (Illumina) according to the manufacturer’s instructions. After cluster generation, the libraries were sequenced on the Illumina HiSeq 4000 platform, and 150-bp paired-end reads were generated.

### Differential Expression Analysis of circRNAs, miRNAs, and mRNAs

Differential expression analysis of two subjects was performed using the DESeq R package (1.10.1). Transcripts with *P* values < 0.05 and |fold change| ≥ 1.5 were considered differentially expressed (DE). The DE RNAs were visualized on volcano plots. A heat map was generated to exhibit hierarchical cluster expression patterns in subjects. The raw counts were normalized using TPM.

### circRNA-Related ceRNA Network Construction

To construct the circRNA-related ceRNA networks, prediction of miRNA-targeted mRNAs, miRNA-targeted circRNAs using the principle of miRNA interference or repression of target genes. The correlation coefficients of miRNA and mRNA were calculated, and a negative correlation was selected; the correlation coefficients of miRNA and circRNA were calculated, and a negative correlation was selected. Based on the results of ceRNA, the mRNAs and circRNAs co-regulated by miRNAs were selected. MicroRNA target sites of circRNAs and mRNAs were identified using miRanda. circRNA-miRNA-gene networks were constructed by Cytoscape software (version 3.6.0).

### Protein–Protein Interaction (PPI) Analyses

The list of differential genes generated in the differential analysis was used to find out the interactions between these differential genes in the STRING database, and the obtained interactions data were imported into Cytoscape software (version 3.6.0) to realize the interactions.

### circRNA Translation Prediction

IRESfinder (Zhao et al., 2018) was used to identify the RNA internal ribosome entry site of circRNAs, including the circRNA splicing sequences and junction sequences based on RNA sequencing (RNA-Seq). Additionally, validated the prediction results by IRESite^1^ (Mokrejs et al., 2010) and CircInteractome^2^ (Dudekula et al., 2016). ORFfinder^3^ was used to predict the open reading frame (ORF) of circRNAs.

### Validation of RNA-Seq Results by qPCR

Total RNA was extracted and purified from plasma exosomes using the miRNeasy Mini kit (Qiagen, DUS, Germany) according to the standard protocol. MiRNAs were reverse-transcribed using the Mir-X miRNA quantitative real-time PCR (qRT-PCR) SYBR Kit (Takara, Japan), and circRNAs and mRNAs were reverse-transcribed using the PrimeScript RT reagent kit (Takara, Japan) and quantitatively amplified by TB-Green Advantage qPCR Premix (Takara, Japan). The relative expression levels of circRNA, miRNA, and mRNA were normalized to the expression levels of *ACTB* and U6 and were determined using the following formula:

$$2^{-\Delta \Delta Ct}$$

The primer sequences are listed in Supplementary Table 1.

### Statistical Analysis

Categorical variables were presented as percentages and analyzed using chi-square tests. Continuous variables were presented as the mean ± standard error of the mean (SEM) or median (interquartile range). Continuous variables were analyzed using independent-samples *t*-tests (normal distribution) or Kruskal-Wallis tests (abnormal distribution). Statistical analyses were performed using SPSS 22.0, and statistical significance was set at *p* < 0.05.

The Predictive Model was constructed by Logistic Regression, and Discrimination and Calibration metrics were used to evaluate the Predictive Model. Among them, receiver operating characteristic (ROC) curves were generated, and the area under the curve (AUC) was calculated to assess the diagnostic value of circRNA expression for discriminating LAA; and the Hosmer-Lemeshow goodness-of-fit test to evaluate the calibration ability of the prediction model.

## Results

### Characterizations of Exosomes Isolated From Peripheral Blood

**FIGURE 1:**
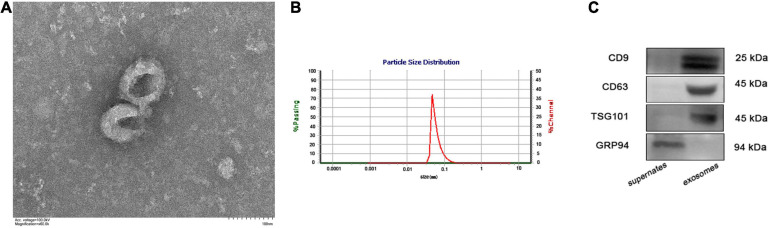
Characteristics of exosomes isolated from peripheral plasma. **(A)** Representative TEM micrograph of exosomes (scale bar of 100 nm). **(B)** NTA analysis of exosomes enriched from plasma indicated they were approximately 50–150 nm in diameter. **(C)** Western blotting showing the protein expression of three positive protein markers (CD9, CD63, and TSG101) and a negative protein marker (GRP94) of exosomes.

A total of 37 LAA patients and 37 normal controls (NCs) were recruited in this study, including five pairs for RNA-Seq and 32 pairs for further verification. The clinical characteristics of the participants included in the discovery cohort are summarized in Supplementary Table 2. Exosomes were isolated from the plasma of all subjects; we used TEM, NTA and western blotting to further verify the isolated exosomes. The morphology of the exosomes was examined by TEM, and a typical image of exosomes is shown in Figure 1A, as ovals without nuclei. The size distribution of the exosomes was evaluated with NTA (Figure 1B). The isolated exosomes ranged between 50 and 150 nm. Western blotting showed that three positive protein markers (CD9, CD63, and TSG101) associated with exosomes were all detected. In contrast, GRP94, a negative marker of exosomes, was absent in isolated exosomes (Figure 1C).

### Expression Profiles of Exosome-Derived circRNAs

**FIGURE 2:**
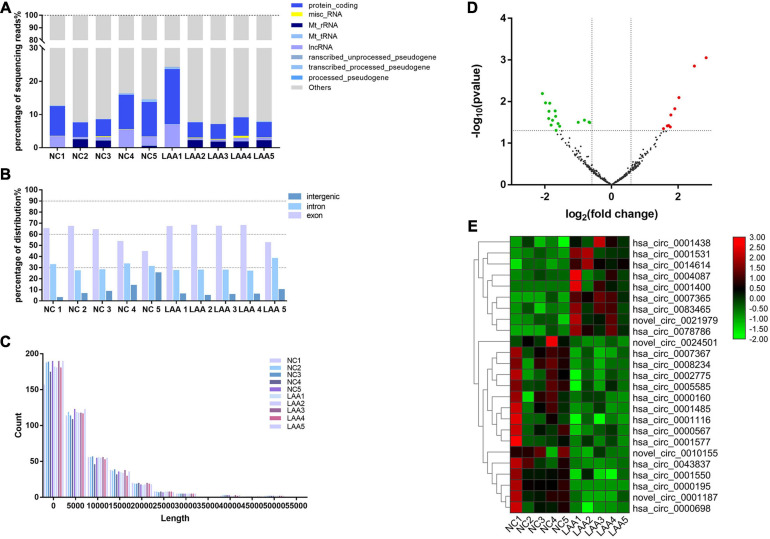
Expression profiles of exosome-circRNAs from RNA-Seq. **(A)** Various RNA biotypes expressed as a percentage of the total sequencing reads by taking the average reads of each biotype across each group. **(B)** circRNAs from exons, introns, or intergenic regions. **(C)** The lengths of circRNAs in each subject. **(D)** Volcano plots of differentially expressed circRNAs. The red and green dots indicate upregulated and downregulated circRNAs, respectively. **(E)** Heat maps of differentially expressed circRNAs.

To obtain a global profile of the exosome-enriched fraction-derived circRNA from the peripheral circulation of LAA stroke patients, we first characterized 10 subjects (5 LAA, 5 NC) using RNA-Seq analysis. HTSeq software was used to analyze the coverage of different known gene types of the species samples using the UNION model. Using the expression-level statistics of the expression distribution of each type of gene in the sample, the distribution of known gene types was obtained, as shown in Figure 2A, including rRNA, tRNA, and mRNAs. circRNAs were detected and identified using Find circ and CIRI2 (at least one back-spliced read) (Memczak et al., 2013; Gao et al., 2018). circRNAs can be derived from splicing of exons or introns. The circRNA sources were mainly gene exons (Figure 2B), and the lengths of the circRNAs were mostly <2000 bp (Figure 2C).

In this study, a total of 462 circRNAs were detected in exosomes, including NC and LAA subjects; among them, 53 circRNAs were newly discovered. The majority of circRNA species detected in NC exosomes were also detected in LAA exosomes at similar expression levels. We further identified DE circRNAs by performing bioinformatics comparisons between the two groups. 25 DE circRNAs were identified between the NC and LAA subjects. Among the DE circRNAs, nine circRNAs were significantly upregulated and 16 circRNAs were significantly downregulated in the LAA group (Figure 2D). We then used cluster heat map analysis of the DE circRNAs to better distinguish their expression patterns (Figure 2E).

### Go and KEGG Pathways of Exosome-Derived circRNAs

**FIGURE 3:**
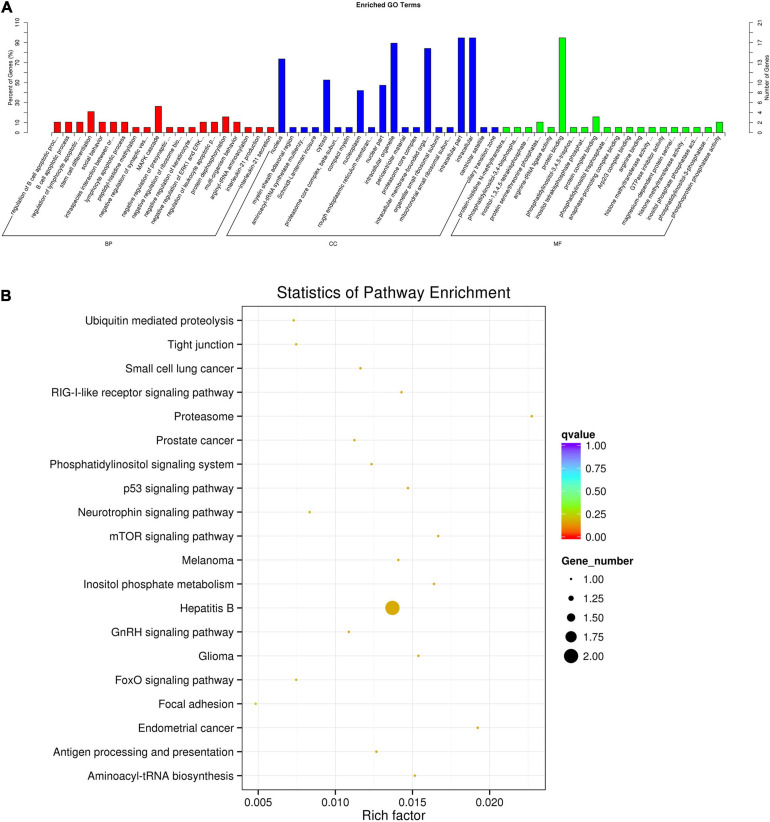
Functional enrichment of the source genes of the DE circRNAs. **(A)** Top 20 biological processes, cell components, and molecular functions according to GO analysis. **(B)** Top 20 pathways according to KEGG analysis visualized on a bulb map. The color of the dot corresponds to different *p-*value ranges, and the size of the dot indicates the number of genes in the pathway.

To screen the potential functions of DE plasma exosome-enriched circRNAs, we analyzed the source gene functions of circRNAs by performing Gene Ontology (GO) and KEGG enrichment analyses. In Figure 3A, the top 20 significant biological processes, cell components, and molecular functions involved in inflammation and immune biological processes are shown according to GO analysis results. Among the top 20 enriched pathways according to KEGG analysis, as shown in Figure 3B, were those primarily related to the regulation of bacterial invasion of the inflammation pathway, such as the mTOR signaling pathway, suggesting that these pathways are involved in the regulation of the LAA stroke process.

### circRNA-miRNA-mRNA ceRNA Network Construction

**FIGURE 4:**
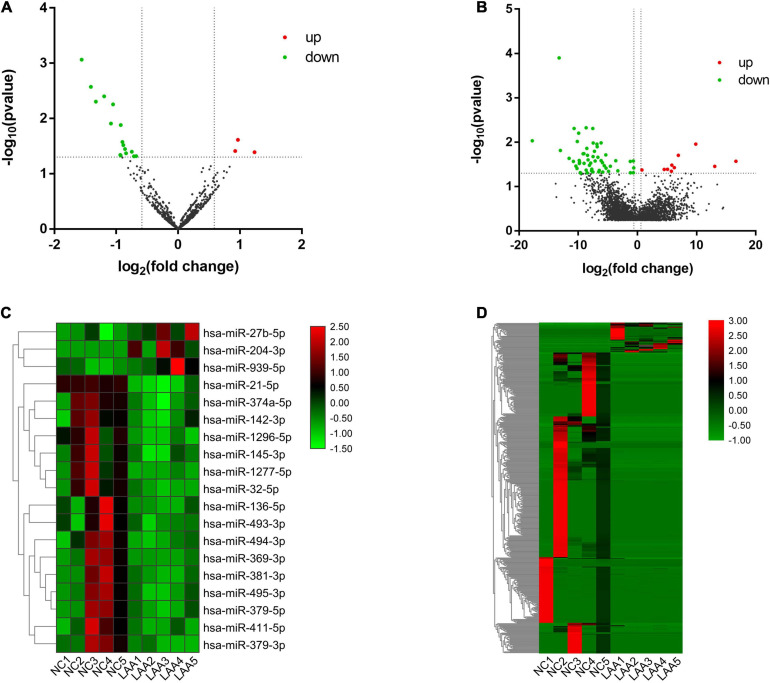
Differential expression and hierarchical cluster analysis of miRNAs, and mRNAs. Volcano plots of significantly differentially expressed RNAs. The red and green dots indicate upregulated and downregulated genes, respectively. The horizontal dotted lines represent a *p*-value of 0.05, and the vertical dotted lines represent a fold change of 1.5-fold. Heat maps of the RNA expression hierarchical patterns. **(A,B)** Volcano plots of differentially expressed miRNAs, mRNAs. **(C,D)** Heat maps of differentially expressed miRNAs, mRNAs.

**FIGURE 5:**
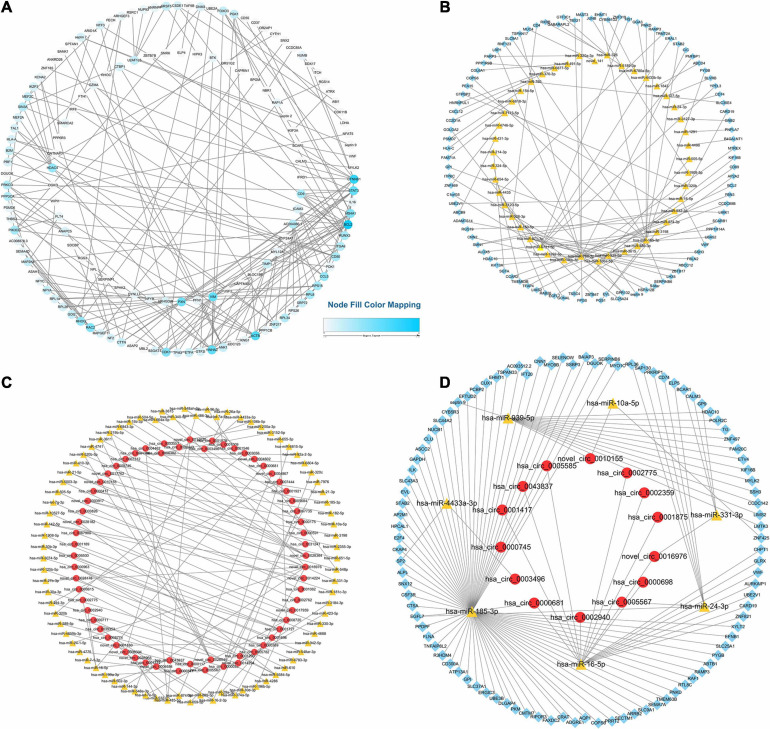
Protein–Protein Interaction and circRNA-miRNA-mRNA network. **(A)** PPI Networks of DE exosomal mRNAs. **(B)** Exosomal miRNA-mRNA regulatory networks. **(C)** Exosomal circRNA-miRNA regulatory networks. **(D)** circRNA-miRNA-mRNA regulatory network. The yellow triangles represent miRNAs, red nodes circRNAs, and blue frames mRNAs.

We further analyzed the potential function of exosomal circRNAs, including miRNA sponges of target miRNAs (Li et al., 2018). We sequenced exosomal miRNA and mRNA directly, and the differential expression of exosome-derived miRNAs and mRNAs between two subjects were analyzed. The results showed that there were 3 upregulated exosomal miRNAs and 16 downregulated exosomal miRNAs and 37 upregulated exosomal mRNAs and 356 downregulated exosomal mRNAs (Figures 4A,B). The hierarchical cluster analysis of the DE miRNAs and mRNAs was performed to better understand their potential relationship (Figures 4C,D). Then, we performed PPI analysis of DE mRNAs to identify important hub genes among LAA stroke (Figure 5A).

Based on ceRNA theory, we looked for miRNA-mRNA pairs with the same miRNA binding sites and established a miRNA-mRNA network. The top 100 miRNA-mRNA pairs based on Pearson correlation coefficients are presented in Figure 5B. Meanwhile, we constructed the miRNA-circRNA network (Figure 5C). Based on the obtained miRNA-mRNA and miRNA-circRNA pairs, an interaction circRNA-miRNA-mRNA network was established. As shown in Figure 5D, different shapes represent different types of RNA, and nodes are enriched to relatively more points in the network and could be more relevant to the biological problem studied. For example, has-miR-393, hsa-miR-16, and hsa-miR-185.

**FIGURE 6:**
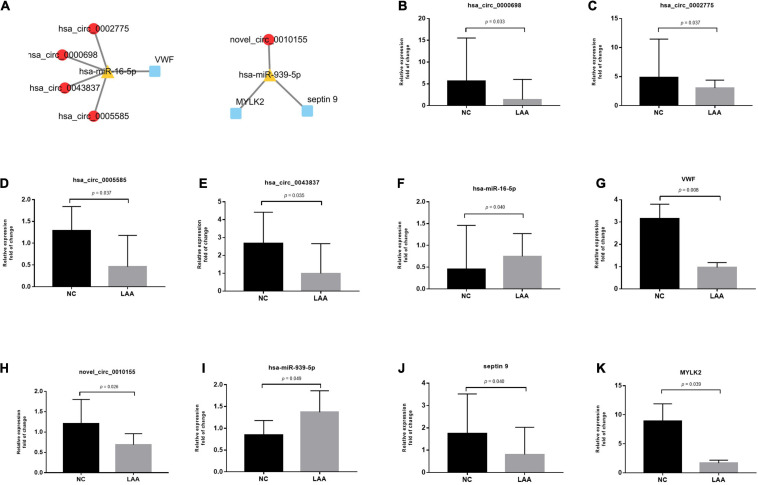
Representative circRNA-miRNA-mRNA network expression validation by qRT-PCR. **(A)** Representative circRNA-miRNA-mRNA network. **(B–G)** The fold changes of LAA and normal subjects’ expression values of hsa_circ_0000698, hsa_circ_0002775, hsa_circ_0005585, hsa_circ_0043837, hsa-miR-16, and VWF. **(H–K)** The fold changes of the LAA and normal subjects’ expression values of novel_circ_0010155, has-miR-393, septin9, and MYLK2.

In the validation phase, we selected 2 of the constructed ceRNA networks for validation, mainly based on differential expression and functional enrichment of genes (Figure 6A). To verify the predicted ceRNA networks, the RNA expression levels in the circRNA-miRNA-mRNA networks were validated by real-time PCR in both LAA and NC subjects (LAA: NC = 32: 32). In Figures 6B–G, hsa_circ_0000698, hsa_circ_0002775, hsa_circ_0005585, hsa_circ_0043837, and VWF were significantly downregulated, and hsa-miR-16 was significantly upregulated. Meanwhile, novel_circ_0010155, septin 9 and MYLK2 were significantly downregulated, and hsa-miR-939 was significantly upregulated between the two groups (Figures 6H–K).

### Translation Potential of Exosomal circRNAs

**FIGURE 7:**
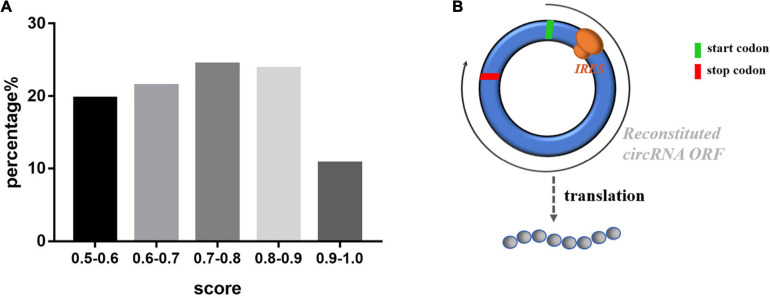
The exosomal circRNAs IRES scores and translation mode pattern. **(A)** Percentage distribution of circRNA IRES prediction scores. **(B)** The Exosomal circRNAs translation mode pattern.

As a member of the noncoding RNA family, circRNAs were previously thought not to be capable of coding because it lacks the 5′ caps and 3′ polyA tails. Recent studies have reported that circRNAs can be translated in a cap-independent manner via sequences that act as internal ribosome entry sites (IRESs) of circRNAs to promote the direct binding of initiation factors or the ribosome to translatable circRNAs (Legnini et al., 2017). We used IRESfinder software, IRESite and CircInteractome to determine whether exosomal circRNAs containing IRES among circRNAs and junction sequences with coding potential. Among them, 381 sequences were identified in the IRES, including 322 junction sequences of exosomal circRNAs. The percentage of IRES scores is shown in Figure 7A, and the top 10 scores of sequences are listed in Table 1. Furthermore, we used ORF Finder to predict the ORFs of the circRNAs. The potential amino acid sequences encoded by the novel exosomal circRNAs in Top10 are shown in Supplementary Table 3. A graph of the possible patterns encoded by circRNA is shown in Figure 7B.

**TABLE 1:** Top 10 score sequences according to IRES prediction.

ID	Index	Score
hsa_circ_0060238_junction_seq	IRES	0.9882155
hsa_circ_0008155_junction_seq	IRES	0.9873013
hsa_circ_0002458_junction_seq	IRES	0.9872067
hsa_circ_0002484_junction_seq	IRES	0.9851023
novel_circ_0010155_junction_seq	IRES	0.9834315
novel_circ_0003849_junction_seq	IRES	0.9833815
hsa_circ_0006845_junction_seq	IRES	0.9831576
hsa_circ_0005616_junction_seq	IRES	0.9809732
novel_circ_0001180_junction_seq	IRES	0.980809
hsa_circ_0034972_junction_seq	IRES	0.9800741

### ROC Curve Analysis of Circulating Exosomal circRNAs in LAA Stroke

**FIGURE 8:**
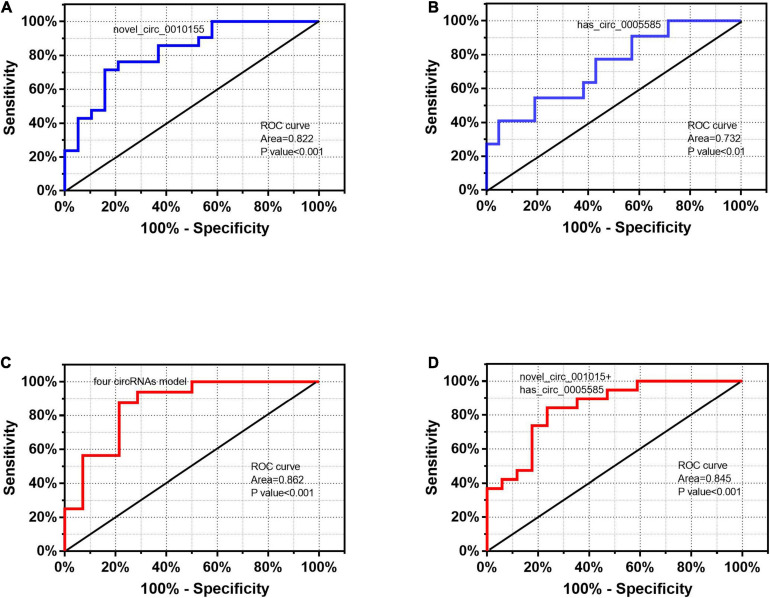
Performance of exosomal circRNAs according to ROC curve analysis. ROC curves of exosome-derived **(A)** novel_circ_0010155, **(B)** hsa_circ_0005585, **(C)** the four circRNA model, and **(D)** novel_circ_001015 plus hsa_circ_0005585.

According to the differential expression and functional prediction, we further screened the circRNAs associated with LAA stroke and predicted the diagnostic value by ROC curve analysis. The expression levels of five circRNAs (novel_circ_0010155, hsa_circ_0005585, hsa_circ_0000698, and hsa_circ_0002775, hsa_circ_0043837) in the ceRNA network were confirmed by real-time PCR. We further selected novel_circ_001015 and hsa_circ_0005585 for ROC diagnostic analysis after constructing logistic regression models for them. The AUC of novel_circ_001015 was 0.822, while that of hsa_circ_0005585 was 0.732 (Figures 8A,B).

Additionally, we combined these candidate exosomal circRNAs using a logistic model with four circRNAs (hsa_circ_0005585, hsa_circ_0000698, and hsa_circ_0002775, hsa_circ_0043837) that exhibited a significant AUC of 0.862 (Figure 8C); novel_circ_001015 plus hsa_circ_0005585 exhibited an AUC of up to 0.845, which was better than that of individual circRNAs (Figure 8D).

## Discussion

In this study, we first analyzed the comprehensive expression profiles of circulating exosomal circRNAs in LAA stroke. Furthermore, we performed the potential functions of exosomal circRNAs: ceRNA network construction and encoding proteins. Importantly, ROC curve analysis revealed that the circulating exosomal circRNAs have potential diagnostic efficacy for LAA stroke.

In the present experiment, we directly performed the sequencing of plasma exosomal circRNA, miRNA and mRNA. Previous studies have found that exosomal RNAs may be protected from degradation by blood-derived ribonucleases, which are more abundant and stable in exosomes (Théry, 2015; Bai et al., 2019; Wang et al., 2019). And a study found that the diagnostic efficacy of exosomal miRNAs in colon cancer is better than plasma (Min et al., 2019). Therefore, our study focuses on the protective role of exosomes for material transport while directly sequencing circulating exosomes for research. In addition, the researchers have demonstrated that exosomes are involved in cell-to-cell communication (Théry et al., 2018; Shi et al., 2020), and endothelial cells, macrophage-derived exosomes are involved in the AS process with crosstalk between different cell types (Tang et al., 2016; Gao et al., 2020). Several scholars, including Tao-Tao Tang (Tang et al., 2020), have conducted mass spectrometry analysis of membrane proteins of exosomal vesicles to search for members of the adhesion protein family for proteins that may be involved in target binding. Based on our comprehensive analysis of exosomal circRNAs, we will investigate the targeting role of circulating exosomes in the LAA mechanism deeply in the following experiments.

Here, a total of 25 exosomal circRNAs were identified as DE between the NC and LAA groups, of which 9 circRNAs were increased and 16 circRNAs decreased in the LAA group. Precious studies have been demonstrated that circRNA plays an important role in the process of ischemic stroke. Clinical studies have reported differences in circRNA compared to controls in acute carotid-related ischemic stroke events, which can be used as a biomarker for ischemic stroke (Zuo et al., 2020). This experiment further investigated the expression profile of circRNA in exosomes of LAA stroke. In validation phase, our results showed that the AUCs of the plasma exosome-derived novel_circ_0010155 and hsa_circ_0005585 were associated with LAA. ROC curve analysis revealed that the above DE exosomal circRNAs could be used to discriminate LAA cases from controls and might thus act as predictive biomarkers. However, this study is subject to the problems of small sample size, and unavoidable selective bias.

Furthermore, GO and KEGG pathway enrichment of DE exosomal circRNAs source genes involved several biological processes such as inflammation, immunity and apoptosis. As mentioned in previous studies, LAA stroke is one of the important subtypes of acute ischemic stroke, which is closely related to AS (Capodanno et al., 2016). And AS is a chronic inflammatory disease that is characterized by endothelial cell dysfunction and monocyte or macrophage accumulation (Libby et al., 2011). Among the KEGG pathway enrichment, circRNAs are associated with classical p53 signaling pathway, mTOR signaling pathway, focal adhesion, etc. mTOR is involved in several cellular processes such as protein synthesis, autophagy and senescence; and mTOR inhibitors have antiatherosclerotic effects (Kurdi et al., 2018). All of the above suggests that the DE exosomal circRNAs are involved in LAA stroke development.

Based on ceRNA theory, we constructed an exosomal circRNA-miRNA-mRNA ceRNA regulatory network. Prior studies that circRNAs have miRNA binding sites, known as miRNA response elements, and can modulate the activity of miRNAs and the downstream-targeted mRNAs of miRNAs (Salmena et al., 2011). Furthermore, in our experiments, we selected two ceRNA pathways for validation based on the following two conditions: first, differential expression of circRNA, miRNA and mRNA; second, functional enrichment of circRNA-derived genes and mRNA. These results were further confirmed using qRT-PCR in both the LAA and NC subjects (LAA: NC = 32: 32), which showed approximately similar trends in RNA-Seq. The accuracy of the prediction was confirmed. The related miRNAs (has-miR-393 and hsa-miR-16) and mRNAs (septin9, MYLK2, and VWF) of the above five circRNAs were revealed to have potential roles in inflammation and cancer. For example, researchers have found that overexpression of miR-16 inhibits the formation of foam cells (Wang M. et al., 2020), suggesting that the target circRNAs are involved in the process of AS.

In addition, our experiments also predicted the coding ability of exosomal circRNAs, mainly including IRES and ORF two components. Emerging evidence also suggests a potential role of circRNAs in translation (Conn et al., 2015; Li et al., 2018). Initial experiments suggested that circRNAs, noncoding RNAs, and ribosomes cannot be loaded onto circRNAs due to their lack of a 5′ end 7-methylguanosine (m7G) cap, but researchers have reported that circRNAs can be translated via an IRES, allowing synthetic circRNAs to be translated in a cap-independent manner (Yang et al., 2017). Based on the IRESfinder software prediction, we then validated the prediction results by IRESite and CircInteractome. The IRESfinder model we used to be Zhao’s model for predicting IRES probabilities, based on experimentally validated IRESs, which are more efficient for prediction (Wang and Gribskov, 2019). Also, we will verify the predicted coding potential of exosomal circRNAs and the mechanism of the generated peptides in LAA stroke in further studies.

In conclusion, our study is the first to describe the comprehensive expression of exosomal circRNAs in LLA stroke. Importantly, circRNA ceRNA networks and translatable analysis revealed their miRNA sponges and encoding proteins biological functions in LAA progression. Our study may provide the potential diagnostic and biological functions for exo-circRNAs of LAA in future studies.

## Data Availability Statement

The data presented in the study are deposited in the Gene Expression Omnibus database (https://www.ncbi.nlm.nih.gov/geo/), accession number (GSE173719).

## Ethics Statement

The studies involving human participants were reviewed and approved by the Ethical Committee of the Affiliated Hospital of Qingdao University. The patients/participants provided their written informed consent to participate in this study.

## Author Contributions

All authors read and approved the final version of the manuscript. XZ, XP, and AM were involved in the study design. QX, RY, YW, and SY collected the samples, performed the experiments, and analyzed the data. QX and XZ completed the manuscript.

## Conflict of Interest

The authors declare that the research was conducted in the absence of any commercial or financial relationships that could be construed as a potential conflict of interest.

## Footnotes

1 http://www.iresite.org/

2 https://circinteractome.nia.nih.gov/

3 https://www.ncbi.nlm.nih.gov/orffinder/
