# Supplementary material for: Comprehensive Analysis of Peripheral Exosomal circRNAs in Large Artery Atherosclerotic Stroke
Source: Front Cell Dev Biol. 2021 Jun 21;9:685741. doi: 10.3389/fcell.2021.685741 (PMC8257506; doi:10.3389/fcell.2021.685741)
Supplement: Supplementary file 1 [file Table_1.DOCX]

**Supplementary Table S1. The primer sequences of qRT-PCR**

| RNAs | Primer Sequences |
| --- | --- |
| novel_circ_0010155 | Forward CAGGGCCCAGGGAAATGAGA  Reverse GCCTCTCATCCTTGTCGTCTGT |
| hsa_circ_0000698 | Forward CCTCAGGACTCCAGATTATCTACAACA  Reverse ACAGCAGCAATGTTGACTTGACA |
| hsa_circ_0002775 | Forward CTGACACGAATGCGCACTGA  Reverse TGACCAGCATCCTTGGAGCT |
| hsa_circ_0005585 | Forward AAACATGTGACGCGCTCCAG  Reverse CCAGCTGTTGAAGTGGTGCC |
| hsa_circ_0043837 | Forward TGGTCCTTCGCCGTCAGTAT  Reverse ACAGACTCAGGCTGACTCCA |
| hsa-miR-939-5p | TGGGGAGCTGAGGCTCTG |
| hsa-miR-16-5p | CGCTAGCAGCACGTAAATATTGGCG |
| septin 9 | Forward TCCACTGCTGCCTCTACTTCATCC  Reverse GGACGATGTTGACCACCTTGCTC |
| MYLK2 | Forward CAGGGAAGACAGAGCAGGCTTTG  Reverse GTGTTGGTGGTCGCAAGGAAGG |
| VWF | Forward CCTGTTACTATGACGGTGAGAT  Reverse CATGAAGCCATCCTCACAGTAG |

**Supplementary Table S2. Clinical characteristics of LAA patients and control subjects**

| Variables | LAA groups  (*Mean*±SEM) | NC groups  (*Mean*±SEM) | *P* value |
| --- | --- | --- | --- |
| Ages (years) | 66.29±2.11 | 60.88±1.98 | 0.896 |
| Sex (man, %) | 22 (59.5%) | 19 (51.4%) | 0.640 |
| BMI (kg/m^2^) | 23.24±0.92 | 25.18±1.24 | 0.640 |
| Hypertension (n, %) | 20 (54.1%) | 13 (35.1%) | 0.160 |
| Diabetes (n, %) | 6 (16.2%) | 6 (16.2%) | 0.623 |
| Smoking (n, %) | 19 (51.4%) | 10 (27.0%) | 0.056 |
| Drinking (n, %) | 16 (43.2%) | 9 (24.3%) | 0.140 |
| TC (mmol/L) | 4.10±0.21 | 4.09±0.22 | 0.265 |
| TG (mmol/L) | 1.18±0.13 | 1.49±0.21 | 0.068 |
| LDL (mmol/L) | 2.49±0.18 | 2.32±0.16 | 0.088 |
| HDL (mmol/L) | 1.20±0.09 | 1.17±0.84 | 0.639 |
| Basal NIHSS | 4.78±1.00 | 0 | <0.0001* |

**Supplementary Table S3. Novel circRNAs junction sequences and ORFs of Top 10 IRES prediction**

| **ID** | **Junction Sequence** | **ORF \| aa** |
| --- | --- | --- |
| novel_circ_0010155 | TAACCTTTTTTTTTTTTTTTTTGGCAGGGTAGGTGAGAGTGAATACAACAGACGAC | MLQFHTKSRAQTTAAHHPDSGLNDMSHCYLWVIPRHERHITWFVGSTICSNLFYKQHERRGRESHLLGDGCKDMSQGLWWSSPRQESLIQYKLGPALGHDIQHMQGPGK |
| novel_circ_0003849 | TTTAGAAACTTCTTTACAATATTAAATATGTAAGTAATAGATTGAAATTTTGAAAT | MKINFKISIYYLHNIVKKFLKMGKILSLQ |
| novel_circ_0001180 | CTTATTTTCTCTTAATTTCTATTTGAAGGATATGGAGTTTGGACCTCCAACAGTTA | MWKLLLSVNTMFYDINIYTLFSLNFYLKDMEFGPPTVNDKLMRFFDHCEK  FLTEVEKNATALYHVEAFKTGPEMQNILKKVAATLQVPVNDLNAGNMSVV |
